# Identifying individuals with attention deficit hyperactivity disorder based on temporal variability of dynamic functional connectivity

**DOI:** 10.1038/s41598-018-30308-w

**Published:** 2018-08-07

**Authors:** Xun-Heng Wang, Yun Jiao, Lihua Li

**Affiliations:** 1 College of Life Information Science and Instrument Engineering, Hangzhou Dianzi University, Hangzhou, 310018 China; 2 Jiangsu Key Laboratory of Molecular and Functional Imaging, Department of Radiology, Zhongda Hospital, Medical School of Southeast University, Nanjing, 210009 China

## Abstract

Attention deficit hyperactivity disorder (ADHD) is a common disorder that emerges in school-age children. The diagnostic model based on neuroimaging features could be beneficial for ADHD in twofold: identifying individuals with ADHD and discovering the discriminative patterns for patients. The dynamic functional connectivity of ADHD remains unclear. Towards this end, 100 children with ADHD and 140 normal controls were obtained from the ADHD-200 Consortium. The raw features were derived from the temporal variability between intrinsic connectivity networks (ICNs) as well as the demographic and covariate variables. The diagnostic model was based on the support vector machines (SVMs). The performance of diagnostic model was analyzed using leave-one-out cross-validation (LOOCV) and 10-folds cross-validations (CVs). The diagnostic model based on inter-ICN variability outperformed that based on inter-ICN functional connectivity and inter-ICN phase synchrony. The LOOCV achieved total accuracy of 78.75%, the sensitivity of 76%, and the specificity of 80.71%. The 10-folds CVs achieved average prediction accuracy of 75.54% ± 1.34%, average sensitivity of 70.5% ± 2.34%, and average specificity of 77.44% ± 1.47%. In addition, the discriminative patterns for ADHD were discovered using SVMs. The discriminative patterns confirmed with previous findings. In summary, individuals with ADHD could be identified through inter-ICN variability, which could be potential biomarkers for diagnostic model of ADHD.

## Introduction

Attention deficit hyperactivity disorder (ADHD) is a common disorder that spreads in school-age children^1^. According to epidemiological survey, ADHD affects nearly 5–10% of children and 4% of adults^2^. Patients with ADHD always exhibited problematic behaviors (i.e., inattention, impulsivity), academic failure, social dysfunction in their daily life^3^. Therefore, accurate diagnosis of ADHD is beneficial for individuals, as well as their related family and society. Clinical diagnostic models for ADHD were based on behavioral scales, which was subjective during implementation. The structural and functional MRI-based evidences suggested that the brain of ADHD might wire differently form healthy controls^4–7^, thus neuroimaging features might be potential biomarkers for diagnostic model of ADHD. Several neuroimaging-based diagnostic models have been established for ADHD using machine learning^8–18^. The diagnostic models could be beneficial for ADHD in twofold: classification of patients and discovery of ADHD-related discriminative patterns. However, it is a challenging task to classify individuals with ADHD from healthy controls based on brain imaging features. One challenge is the significant high dimensions of features in the diagnostic models. Another challenge is extracting novel features that could discriminate ADHD. On the one hand, the high dimensional features could increase the complexity of diagnostic models. On the other hand, novel features with low dimensions could be beneficial for diagnostic models of ADHD. So far, the dynamic functional connectivity of ADHD remain unexplored.

Intrinsic connectivity networks (ICN) are spatially independent resting state networks, which are intrinsically dynamic and anti-correlated spontaneous brain systems^19,20^. There are about ten to twenty well-established ICNs found by independent component analysis (ICA)^21–23^. The ICNs were related to specific brain functions and even correlated to behavioral symptoms^24^. The ICNs were consistent across different individuals^25^. Thus, the ICNs might be potential biomarkers for brain disorders. The ICN contains two kinds of features: spatial patterns and temporal patterns. The spatial maps of ICNs were reliable across resting state sessions based on ICA and dual regression^26^. The complexity of temporal patterns for ICNs exhibited moderate-to-high test-retest reliability under different scan conditions^27^. However, the spatial patterns of ICN included tens of thousands of features, which could increase the complexity of the diagnostic models. Notably, the temporal patterns of ICNs included appropriate number of features, which could reflect the network-wise brain dynamics. There are two types of ICN-based temporal patterns: univariate features within ICN and bivariate features between ICNs. Altered intra-ICN amplitude of low frequency fluctuations and inter-ICN functional connectivity were found in children with ADHD^28^. Moreover, the intra-ICN entropy and inter-ICN synchrony might predict the clinical symptoms for ADHD^29^. However, the dynamic inter-ICN functional connectivity for ADHD remains largely unexplored.

Dynamic functional connectivity reflected the time-varying properties of brain dynamics. The ICNs exhibited dynamic functional connectivity in healthy subjects^30^. Previous evidence found that there were several dynamical states of functional connectivity between ICNs^31^. Altered dynamic functional connectivity patterns were found between eyes-open and eyes-closed conditions^32^. Moreover, the dynamic functional connectivity might underlie spontaneous fluctuations in attention^33^. Notably, the dynamic functional connectivity could successfully discriminant patients with ADHD^34^. However, those studies focused on the temporal clusters of dynamic functional connectivity. Current evidence found that the strength of functional connectivity showed significant fluctuations over time^35^. Given concerns about the temporal variations in time-resolved ICNs, the temporal variability of dynamic functional connectivity still remains unclear.

In this paper, we aimed to build diagnostic model for ADHD based on temporal variability of dynamic functional connectivity. We also sought to find the discriminative patterns of dynamic functional connectivity for ADHD. To achieve these goals, a cohort of children with ADHD and a cohort of healthy controls were obtained from the ADHD-200 Consortium. The diagnostic model was based on SVMs and inter-ICN variability. The performances of diagnostic model and the most discriminative patterns were determined by leave-one-out cross-validation (LOOCV) and 10-folds CVs, respectively. The prediction accuracy of different inter-ICN features were also analyzed for comparison additionally.

## Results

### Performance of diagnostic models

**Figure 1:**
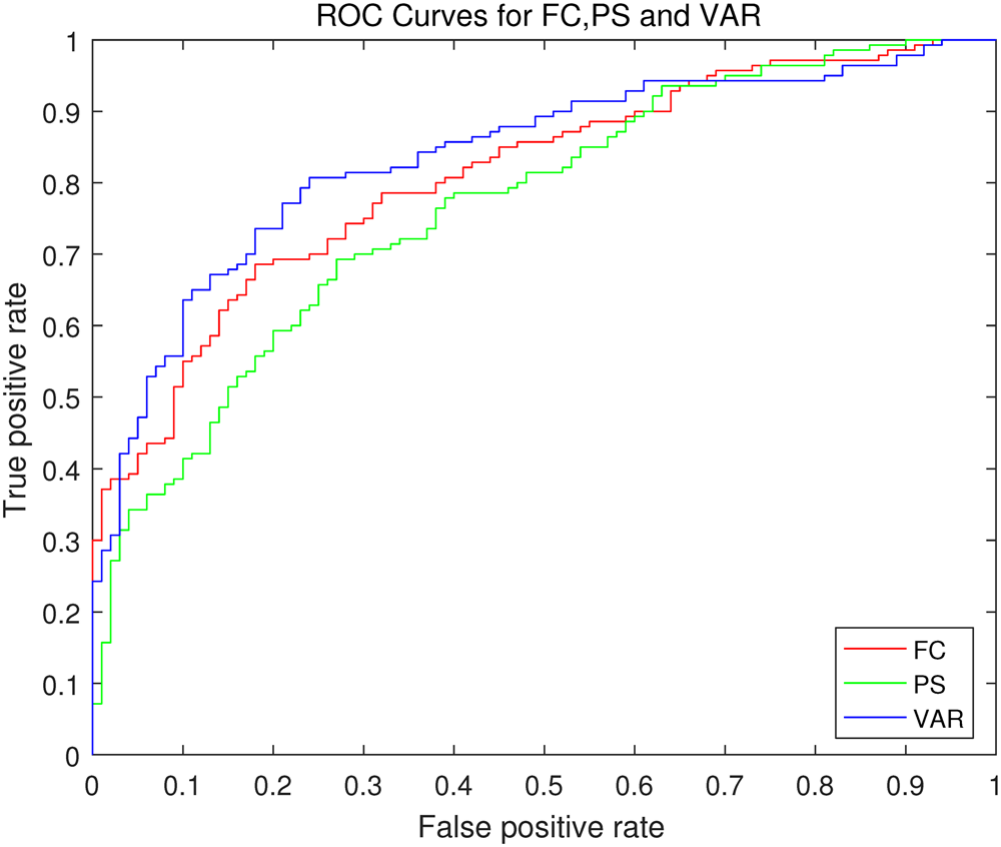
ROC curves based on LOOCV. The red curve denotes ROC based on inter-ICN functional connectivity (FC). The green curve denotes ROC based on inter-ICN phase synchrony (PS). The blue curve denotes ROC based on inter-ICN variability (VAR).

The diagnostic model exhibited moderate performance based on LOOCV. Table 1 shows the performance of LOOCV. Figure 1 shows the receiver operating characteristic (ROC) curves based on different measures. The AUC value based on inter-ICN functional connectivity (FC) is 0.81. The area under curve (AUC) value based on inter-ICN phase synchrony (PS) is 0.77. The AUC value based on inter-ICN variability (VAR) is 0.84. Notably, the diagnostic model based on inter-ICN variability outperforms those based on inter-ICN FC or inter-ICN PS.

**Table 1:** Performance of the diagnostic model based on LOOCV.

performance	accuracy	sensitivity	specificity
VAR	78.75%	76%	80.71%
FC	72.92%	65%	78.57%
PS	68.75%	64%	72.14%

**Figure 2:**
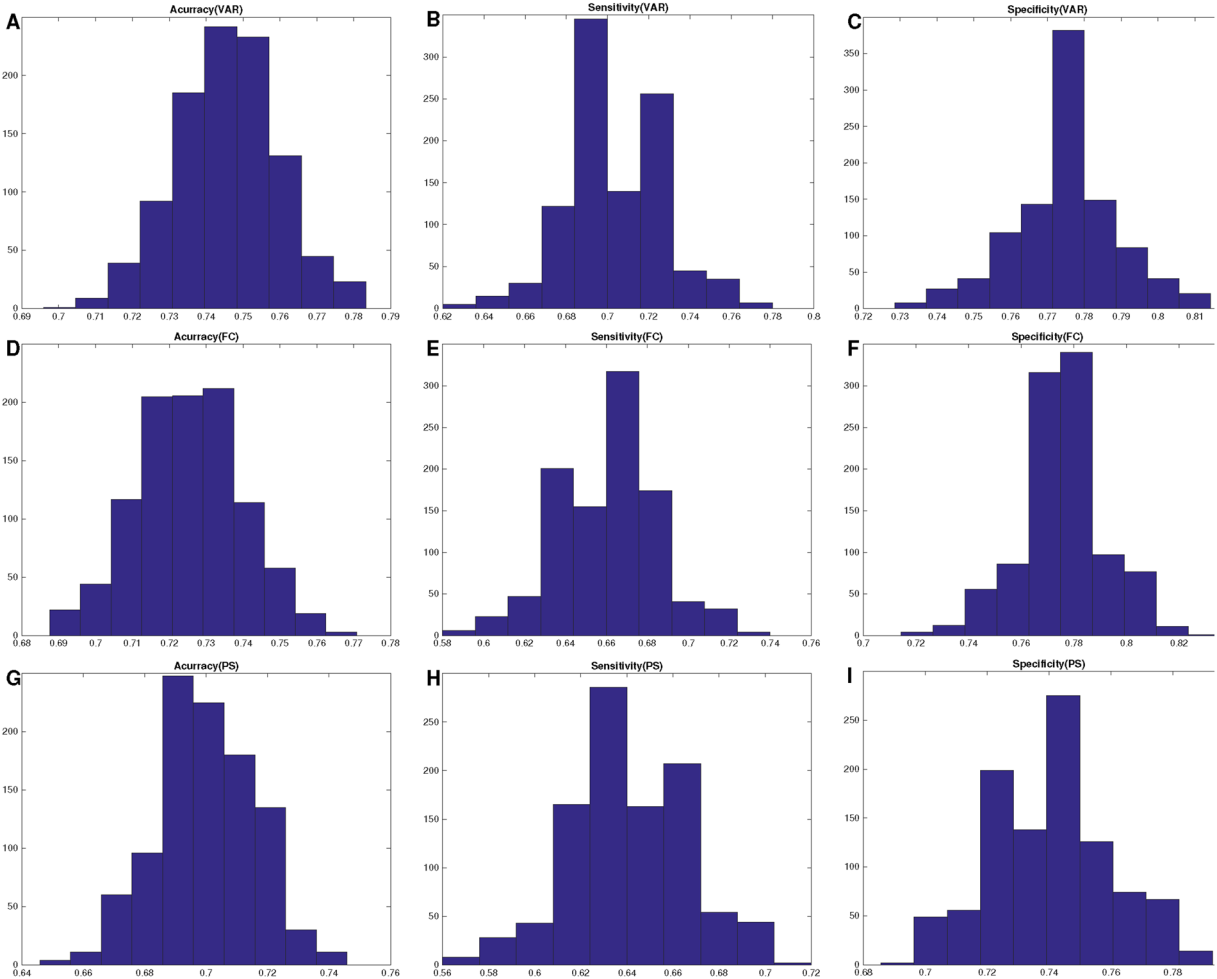
Histograms for performances of 10-folds classifications. Subfigure (**A**) denotes the total accuracy based on inter-ICN variability (VAR). Subfigure (**B**) denotes the sensitivity based on inter-ICN variability. Subfigure (**C**) denotes the specificity based on inter-ICN variability. Subfigure (**D**) denotes the total accuracy based on inter-ICN functional connectivity (FC). Subfigure (**E**) denotes the sensitivity based on inter-ICN FC. Subfigure (**F**) denotes the specificity based on inter-ICN FC. Subfigure (**G**) denotes the total accuracy based on inter-ICN phase synchrony (PS). Subfigure (**H**) denotes the sensitivity based on inter-ICN PS. Subfigure (**I**) denotes the specificity based on inter-ICN PS.

**Figure 3:**
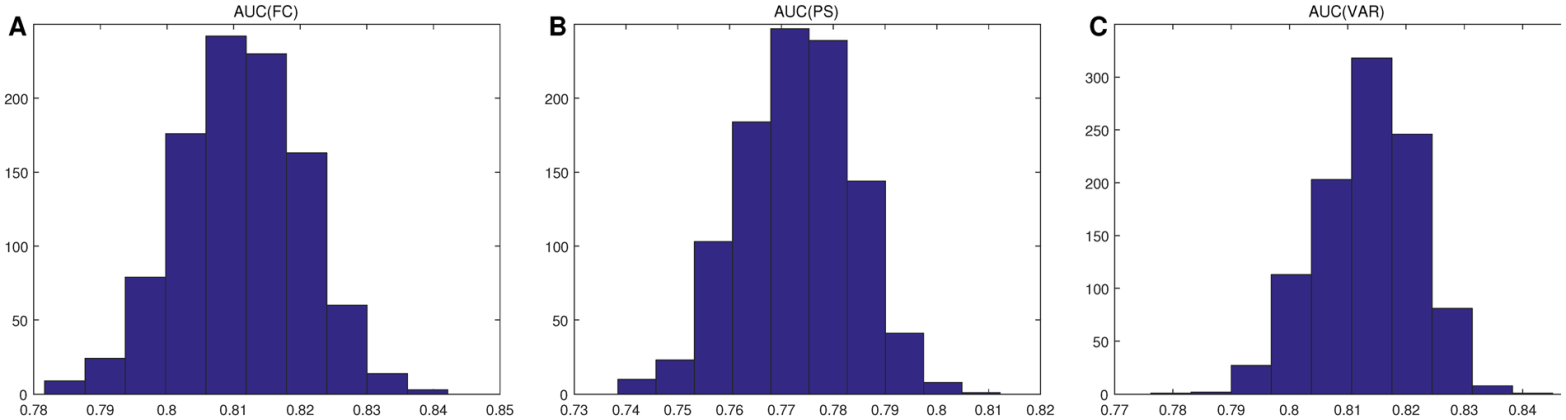
Histograms of AUC values based on 10-folds CV. Subfigure (**A**) denotes histogram of AUC values based on inter-ICN functional connectivity (FC). Subfigure (**B**) denotes histogram of AUC values based on inter-ICN phase synchrony (PS). Subfigure (**C**) denotes histogram of AUC values based on inter-ICN variability (VAR).

The diagnostic model exhibited well-established performance based on 10-folds CVs with 1000 times of simulations. Figure 2 shows the histograms for performances of 10-folds classifications. Figure 3 shows the histograms of AUC values based on different measures. The AUC value based on inter-ICN FC is 0.81 ± 0.01. The AUC value based on inter-ICN PS is 0.77 ± 0.01. The AUC value based on inter-ICN variability is 0.81 ± 0.01. Table 2 show the mean performance of 10-folds classifications. Specially, the diagnostic model based on inter-ICN variability exhibits better performance than those based on inter-ICN FC or inter-ICN PS. Table 3 shows the performance of previous methods and our method.

**Table 2:** Performance of the diagnostic model based on 10-folds CV.

performance	accuracy	sensitivity	specificity
VAR	75.54% ± 1.34%	70.5% ± 2.34%	77.44% ± 1.47%
FC	72.75% ± 1.43%	65.95% ± 2.46%	77.6% ± 1.59%
PS	70.04% ± 1.58%	64.21% ± 2.58%	74.2% ± 1.88%

**Table 3:** Performance of previous methods and our method.

Study	Features	Classifier	Number of features	Subjects (ADHD/TD)	Cross-validation	Acc.	Sen.	Spec.
Zhu *et al*.^8^	Regional Homogeneity	PCA-FDA	>50k	9/11	LOOCV	85%	78%	91%
Wang *et al*.^9^	Regional Homogeneity	SVM	>50k	23/23	LOOCV	80%	87%	74%
Dai *et al*. ^11^	Regional Homogeneity	SVM	>50k	222 /402	10 folds	66%	23%	90%
Colby *et al*.^12^	Multi-modal	SVM	>50k	285/491	10 folds	55%	33%	80%
Cheng *et al*.^13^	Multi-modal	SVM	>50k	101/143	LOOCV	76%	63%	85%
Peng *et al*.^14^	Cortical features	ELM	340	55/55	LOOCV	90%	—	—
Qureshi *et al*.^15^	Cortical features	H-ELM	320	106 /53	10 folds	61%	—	—
Jie *et al*.^18^	Hyper-connectivity	SVM	—	118/98	LOOCV	83%	84%	82%
Present study	Dynamic FC	SVM	50	100/140	LOOCV	79%	76%	81%
Present study	Dynamic FC	SVM	50	100/140	1000 times of 10 folds	76%	71%	77%

### Discriminative patterns for ADHD based on LOOCV

**Figure 4:**
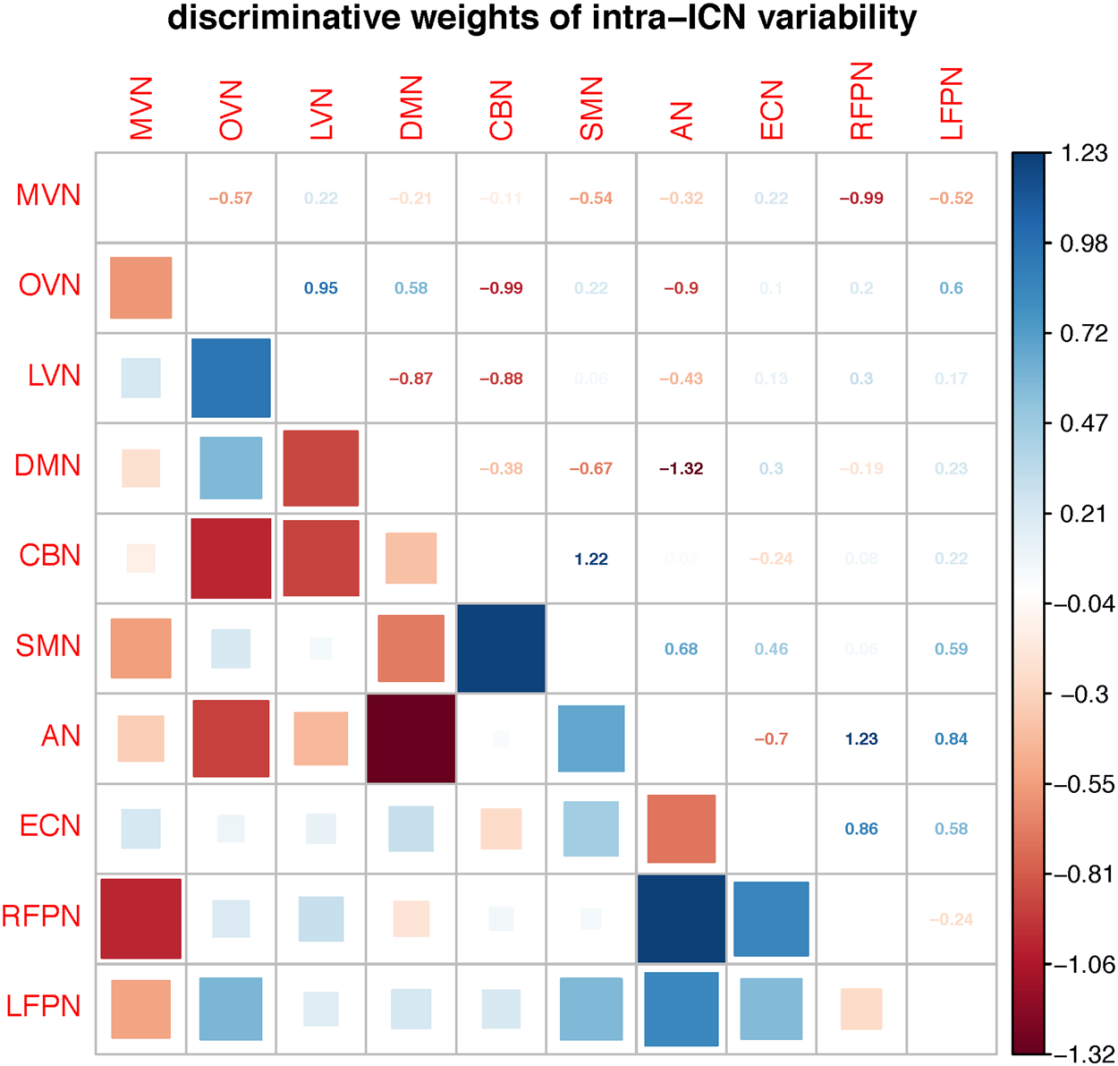
Discriminative patterns based on LOOCV. Red squares denote decreased inter-ICN variability in ADHD. Blue squares denote increased inter-ICN variability in ADHD.

Figure 4 shows the ADHD-related discriminative patterns of inter-ICN variability discovered by LOOCV. There are 19 negative feature-weights and 26 positive feature-weights in the ADHD-related diagnostic model. The power weight of inter-ICN variability between RFPN and AN is 1.23, which is the most positive discriminative weight. The power weight of inter-ICN variability between AN and DMN is −1.32, which is the most negative discriminative weight. Specially, the OVN-related inter-ICN variability exhibits discriminated powers. The power weight of inter-ICN variability between RFPN and MVN is −0.99. The power weight of inter-ICN variability between OVN and LVN is −0.95. The power weight of inter-ICN variability between OVN and CBN is −0.99. The power weight of inter-ICN variability between OVN and AN is −0.9. The LVN also shows discriminative powers. The power weight of inter-ICN variability between LVN and DMN is −0.87. The power weight of inter-ICN variability between LVN and CBN is −0.88. In addition, the power weight of inter-ICN variability between SMN and CBN is 1.22.

### Discriminative patterns for ADHD based on 10-folds CV

**Figure 5:**
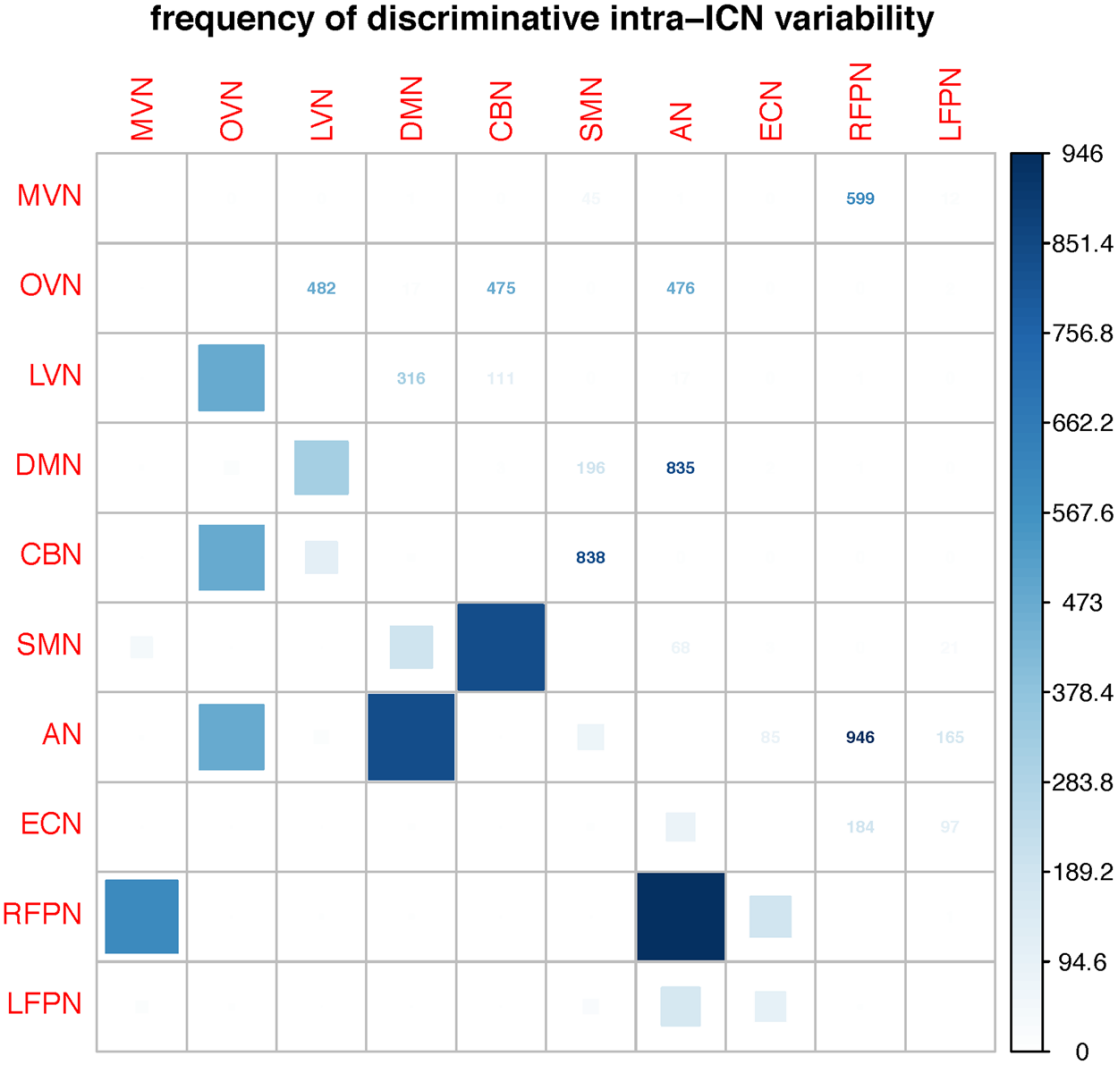
Frequency of discriminative patterns based on 10-folds CVs. The numbers and squares denote the frequency of top 10 discriminative patterns based on 10-folds CV.

Figure 5 shows the frequency of ADHD-related discriminative patterns of inter-ICN variability discovered by 10-folds CVs. The results of frequencies are the top 10 most discriminative features based on 10-folds CVs using 1000 times of simulations. The inter-ICN variability between RFPN and AN appears 946 times. The inter-ICN variability between SMN and CBN appears 838 times. The inter-ICN variability between DMN and AN appears 835 times. The inter-ICN variability between MVN and RFPN appears 599 times. Specially, the OVN exhibits discriminative power during 1000 simulations of 10-folds CVs. The inter-ICN variability between OVN and LVN appears 482 times. The inter-ICN variability between OVN and CBN appears 475 times. The inter-ICN variability between OVN and AN appears 476 times.

### Discriminative weights of demographic and covariate variables

Table 4 shows the discriminative weights of demographic and covariate variables during LOOCV and their frequencies during 1000 simulations of 10-folds CVs. The LOOCV reveals that the discriminative weight for gender is 1.63. The discriminative weight for age is 0.35. Notably, the intelligence quotient (IQ) parameters exhibit discriminative power. The discriminative weight for verbal IQ is −0.73. The discriminative weight for performance IQ is −2.17. The discriminative weight for full IQ is −1.77. In addition, the discriminative weight for head motion is 1.63.

**Table 4:** Discriminative powers of demographic variables.

	LOOCV	10-folds CV
Gender	1.63	1000
Age	0.35	0
Verbal IQ	−0.73	143
Performance IQ	−2.17	1000
Full IQ	−1.77	1000
FD	1.63	994

Moreover, the diagnostic model is simulated 1000 times using 10-folds CVs. Considering the top 10 most discriminative features, the gender parameter appears 862 times. The verbal IQ parameter appears 143 times. The performance IQ parameter appears 1000 times. The full IQ parameter appears 1000 times. In addition, the head motion parameter appears 994 times during 1000 simulations of 10-folds CV.

## Discussions

This paper proposed novel inter-ICN variability to identify individuals with ADHD. To the best of our knowledge, this is the first study that investigated the inter-ICN variability for ADHD. The inter-ICN variability was based on the variance of phase differences. The performance of diagnostic model was based on LOOCV and 10-folds CVs. Specially, the 10-folds CVs was simulated 1000 times to reduce overfitting problems. The well-validated results suggested that the diagnostic model could identify patients with ADHD from normal controls. The discriminative patterns of inter-ICN variability was found using LOOCV and 10-folds CVs. In addition, the IQ and head motion parameters were related to ADHD. Together, ADHD could be discriminated from healthy subjects through the inter-ICN variability as well as demographic and covariate variables. The discriminative patterns of inter-ICN variability could be potential biomarkers for ADHD.

### Performance of diagnostic model

A number of diagnostic models have been built for ADHD based on resting state fMRI. Using a relatively small number of subjects, patients with ADHD could be discriminated from healthy controls based on feature extraction and support vector machines (SVMs)^8,9^. ADHD-200 Consortium provided a significant large number of samples of ADHD and normal controls^10^. Based on ADHD-200 samples, different kinds of features and machine learning methods were applied to diagnosis ADHD^11–13^. Using ADHD-200 samples, the best performance of classification for ADHD is 69.59% based on voxel-wise network features^17^. Total accuracy of 55% was achieved using multimodal features^12^. The prediction accuracy is 67% for combined type and inattentive type of ADHD classification based on combination of functional features^36^. However, most of the current diagnostic models for ADHD exhibited poor performance with either low sensitivity or low specificity. One possible explanation of low prediction accuracy was that the dimensions of neuroimaging features were relatively high in most diagnostic models. The regional cortical surface-based morphological patterns could discriminate ADHD patients from healthy controls based on machine learning methods^14,15^. However, surface-based or voxel-based morphological patterns were structural measures, which could not reflect the brain function or topology of ADHD. Notably, the diagnostic model based on hyper-connectivity could classify ADHD with total accuracy of 82.9%, sensitivity of 83.9% and specificity of 81.6%^18^. However, the performance of classifiers was not validated using sufficient simulations of 10-folds CVs. Moreover, the mathematical implementation of the aforementioned study was more complex than conventional methods. With novel inter-ICN variability, this study could identify patients with ADHD based on LOOCV and 10-folds CVs. Using LOOCV, the total accuracy was 78.75%, the sensitivity was 76%, and specificity was 80.71%. Using 10-folds CVs, the average prediction accuracy was 75.54% ± 1.34%, the average sensitivity was 70.5% ± 2.34%, and the average specificity was 77.44% ± 1.47%. The diagnostic model exhibited balanced accuracies of sensitivity and specificity. Therefore, the diagnostic model could be beneficial for clinical applications. In addition, we proposed a novel neural metrics with relatively low dimensions of features, which could reduce the complexity of the diagnostic models. Specially, the results were based on 1000 times 10-folds CV, while most of the previous methods were based on LOOCV or one run of 10-folds CV. In summary, our method could open a new perspective for diagnosing ADHD.

### Discriminative patterns of dynamic functional connectivity for ADHD

Discriminative patterns of inter-ICN variability were found for ADHD. In the discriminative model, the positive weights indicated the increased inter-ICN variability in ADHD, while the negative weights indicated the decreased inter-ICN variability in ADHD. Most of the discriminative patterns were with positive weights, which suggested increased inter-network activity in ADHD. Previous evidence found that there were enhanced brain activities in ADHD than normal controls^4^. Altered brain topologies were found in ADHD based on graphical measures^37^. Of note, our previous study also found altered brain topologies based on inter-ICN functional connectivity^28^. In this study, the diagnostic model identified certain features of variability between ICNs (i.e., AN-RFPN, AN-DMN) that exhibited discriminative powers. The DMN-related cortex exhibited altered resting state regional homogeneity^9^. Enhanced DMN-related activities were correlated to sustained attention deficits^38^. The RFPN-related variability might be related to the prefrontal-striatal-cerebellar circuit in ADHD^39^. In addition, the visual-related ICNs (i.e., MVN, OVN) were found with discriminative powers. The ICN-based evidences noted that the sensory- and visual-related brain networks might contribute to ADHD^28^. Previous evidence also found enhanced brain activities in sensory-related brain regions^4^. In summary, the informative discriminative patterns could be beneficial to diagnostic model of ADHD.

### Temporal variability of dynamic functional connectivity

The temporal variability of dynamic functional connectivity exhibited better discriminative power than conventional measures of connectivity. A possible explanation was that the inter-ICN variability might reflect the dynamic properties of brain networks. The inter-ICN functional connectivity and phase synchrony was static measures, which contained less information of brain dynamics than inter-ICN variability. Previous evidence suggested that the brain should exhibited dynamic activities during resting state. Seven temporal clusters of dynamic functional connectivity were found based on sliding-window and machine learning methods^31^. Moreover, the dynamic functional connectivity between ICNs were related to visual attention (i.e., eyes-open/closed states)^32^. However, the aforementioned studies were based on temporal clustering, which could not reflect the temporal fluctuations of dynamic functional connectivity. A previous study applied amplitude of low frequency fluctuations (ALFF) on temporal dynamic functional connectivity to predict brain maturity^40^. However, the results were based on sliding-window method, which has certain limitations (i.e., manually selection of sliding-window length). Given current concerns on temporal variability of dynamic functional connectivity, the proposed inter-ICN variability was based on the phase differences, which did not depend on sliding-window. Thus, the inter-ICN variability might be potential biomarkers for human connectome.

### IQ and ADHD

Previous evidences found that the IQ was related to ADHD. The developments of cortical thickness and white matters might be delayed in ADHD with low IQ^41^. The IQ scores also correlated to ALFFs within ICNs, implying that the IQ might contribute to ADHD^28^. A recent study found that functional connectivity between ICNs could predict the IQ scores for ADHD^42^. In this paper, the IQ scores exhibited discriminative power for ADHD, suggesting that the IQ might play an important role in ADHD. Without the IQ scores, the diagnostic model achieved total accuracy of 71.67% based on LOOCV, which was much lower than that with the IQ scores. The personal characteristic features (i.e., IQ) could outperform neuroimaging data in diagnostic models for ADHD^43^. Moreover, the IQ scores were negatively related to ADHD in our diagnostic model. The results implied that the ADHD group exhibited lower IQ scores than healthy group.

### Head motion and ADHD

Given current concerns on head motion in resting state fMRI, the head motion parameter was taken as supplementary feature in the diagnostic model. The power weight of head motion was 1.63, which means that patients exhibited more head motion than normal controls. Without head motion parameter, the diagnostic model achieved total accuracy of 74.17% using LOOCV, which was lower than that with head motion. Thus, ADHD might possess distinctive head motion during resting state. The head motion has been identified as an important confound in resting state fMRI. Previous studies suggested that the subjects with Frame-Displacement (FD) more than 0.5 mm should be discarded^44,45^, since the functional signals were corrupted with severe head motions. The original sample size for this study was 245 subjects. Only 5 subjects exhibited severe head motion, resulting in 240 subjects for diagnostic model. Thus, the preprocessed subjects could represent the original samples. Besides, we used FD as a covariate in the diagnostic models. Previous studies suggested that head motion should be considered as a covariate variable in statistical models^44,45^. Moreover, head motion might affect the test-retest reliability of resting state features^27,46^. For group-wise analysis, the mean FD was computed as head motion parameter^47,48^. In this study, head motion significantly improved the performance of diagnostic model. The results suggested that head motion should be carefully considered in ADHD research.

### Advantages

One advantage of this study was applying inter-ICN variability to diagnosis ADHD. The feature dimensions of inter-ICN variability was much lower than regional- or voxel-wise measures. Moreover, the inter-ICN variability outperformed conventional connectivity estimators in the diagnostic model. Thus, the inter-ICN variability could be beneficial for classification of ADHD. Another advantage of this study was applying 1000 simulations of 10-folds CVs on the diagnostic models. The results were well-validated compared to previous studies. Moreover, the prediction accuracy was balanced with equal sensitivity and specificity. The third advantage of this study was applying SVMs to discover the discriminative patterns for ADHD. The linear SVMs found certain discriminative features, which could be potential biomarkers for ADHD. The fourth advantage of this study was applying the IQ scores as the predictors in the diagnostic models. The IQ scores were related to ADHD, and could significantly improve the performances of the diagnostic models. The fifth advantage of this study was applying head motion as a predictor in the diagnostic models. The head motion parameter was found to be associated with ADHD. The diagnostic models exhibited better performances with head motion parameter. Overall, the inter-ICN variability, IQ scores and head motion might contribute to diagnosis of ADHD. Moreover, the diagnostic models with balanced performances was well-validated using 1000 simulations of 10-folds CVs.

### Limitations

There were several limitations which should be noted in this study. One limitation was that the neuroimaging datasets were obtained using different scan parameters. There were more than three different scan protocols for this dataset. Due to the limited information for this public datasets, we introduced the scan difference (i.e., Peking_1, Peking_2, Peking_3, Peking_1_test) as a covariate. However, the performance of diagnostic model with scan differences was a little lower than that without scan differences. The diagnostic model with scan differences exhibited accuracy of 76.67%, sensitivity of 72%, and specificity of 80%. One possible explanation was that there were five different scan protocols for anatomical images in Peking_3. Thus, the covariate of scan difference might be mislabeled in the diagnostic models. The effects of scan parameters should be taken into account in future study. Another limitation was that the diagnostic model was based on Chinese children in order to remove the effects of populations and versions of clinical scales. We tested our diagnostic model on datasets from the NYU site. However, the diagnostic model only achieved total accuracy of 54.08%. We also applied our method on the NYU children using LOOCV, which achieved total accuracy of 58.67%. One explanation was the training and testing models were based on different populations. Another explanation was the different scan parameters of the two datasets. The third explanation was the two datasets were based on different versions of clinical scales. Therefore, we should test our model on an independent dataset of Chinese children in future study. The parameters of classifiers should be optimized to improve the performance of the diagnostic model. The third limitation was not considering the subtypes in the diagnostic model, since the subtypes contained the label information. The diagnostic model for combined type of ADHD and normal controls exhibited total accuracy of 69.44%, sensitivity of 73.0% and specificity of 65.7%. The diagnostic model for inattentive type of ADHD and normal controls exhibited total accuracy of 77.3%, sensitivity of 77.4% and specificity of 77.1%. The fourth limitation was based on single modal of neuroimaging features. The performance of the diagnostic model could be improved with multi-modal imaging methods (i.e., anatomical MRI, diffusion MRI, arterial spin labeling MRI). The fifth limitation was not considering the eyes-open/closed conditions in the diagnostic model. The eyes-open/closed conditions could affect functional connectivity. The subjects in this study were asked to have their eyes opened or closed. However, we cannot control this confounding variable due to limited information provided by ADHD-200 website. Additionally, the inter-ICN biomarkers should be validated using an independent dataset for clinical approach. Thus, we plan to test the diagnostic model using different features and populations in further study. We also plan to obtain multimodal imaging datasets in further study. Moreover, the performance of the diagnostic model should be improved using different machine learning methods (i.e., extreme learning machines, Bayesian-based classifiers, and neural networks).

## Conclusion

This paper investigated the temporal variability of dynamic functional connectivity to diagnosis children with ADHD based on machine learning methods. The diagnostic models could discriminate patients with ADHD using cross-validations. Moreover, the discriminative patterns of inter-ICN variability were discovered by the diagnostic model. In summary, individuals with ADHD could be identified by machine learning based on the inter-ICN variability, which could be potential biomarkers for ADHD.

## Methods

### Participants and MRI protocols

The ADHD-200 Consortium provided a great number of individuals with ADHD and normal controls (http://fcon_1000.projects.nitrc.org/indi/adhd200/). ADHD-200 Consortium were consisted of datasets from eight sites (i.e., New York University, Peking University, etc.). To remove the effects of sites, the participants in this study were based on datasets from the Peking site. There were 245 participants using datasets combined from Peking_1, Peking_2, Peking_3 and Peking_1_test. The demographic and covariate information could be found in Table 5. The participants were diagnosed using ADHD Rating Scale (ADHD-RS) IV. Participants were selected using the following criteria: 1) right-handedness; 2) without loss of consciousness caused by head trauma; 3) without history of neurological disease and other mental disorder (i.e., schizophrenia, affective disorder, pervasive development disorder, or substance abuse). In addition, the IQ score for each subject was greater than 80 evaluated by Wechsler Intelligence Scale for Chinese Children-Revised (WISCC-R). All research of this study was approved by the Research Ethics Review Board of the Institute of Mental Health, Peking University. The guidelines and regulations of experiments were carried out in accordance with institutional review boards of the Institute of Mental Health, Peking University. Informed consent was provided by the parent of each participant and all of the children agreed to participate in this research.

**Table 5:** Subjects’ demographic and covariate variables.

	ADHD	Normal	*p*-value
Number of subjects	100	140	—
Gender (male: female)	88:12	81:59	<10^−3^
Handless (R: L)	140:0	140:0	1
Age (year)	12.1 ± 2.05	11.44 ± 1.86	0.0093
Verbal IQ	111.44 ± 15.57	120.46 ± 13.3	<10^−5^
Performance IQ	99.03 ± 13.65	111.29 ± 14.33	<10^−9^
Full IQ	106.36 ± 13.02	118.02 ± 12.12	<10^−10^

Both anatomical MRI and resting state fMRI were scanned for each subject. The neuroimaging datasets were obtained from a SIEMENS TRIOTIM syngo 3-T MRI scanner. The anatomical MRI data was a high-resolution T1-weighted MPRAGE 3D volume, which was defaced to protect patient identity. The parameters of the anatomical MRI could be found in Table 6. The resting state fMRI data was a standard T2-weighted EPI 4D volume. The parameters of the functional MRI could be found in Table 7. During each scan, the participants were required to keep relax, stay still with their eyes either open or closed. A black screen with a white fixation cross was presented to each participant during the scan. The additional information could be obtained from the website of ADHD-200 Consortium.

**Table 6:** Scan parameters for anatomical MRIs.

	Peking_1	Peking_2	Peking_3	Peking_1_test
TR/TE (ms)	2530/3.39	2530/3.45	5 protocols	2530/3.39
Slices	128	176	5 protocols	128
Thickness(mm)	1.33	1	5 protocols	1.33
FoV read(mm)	256	256	5 protocols	256
Fov phase	100%	81.3%	5 protocols	100%
Flip angel (degree)	7	7	5 protocols	7

**Table 7:** Scan parameters for functional MRIs.

	Peking_1	Peking_2	Peking_3	Peking_1_test
TR/TE (ms)	2000/30	2000/30	2000/30	2000/30
Slices	33	33	30	33
Thickness(mm)	3.5	3	4.5	3.5
FoV read(mm)	200	200	220	200
Fov phase	100%	100%	—	100%
Flip angel (degree)	90	90	90	90
volumes	240	240	240	239

### Data preprocessing

The anatomical and functional MRI datasets were preprocessed using scripts from 1000 functional connectome project. The preprocessing scripts were based on AFNI and FSL. The anatomical MRI datasets were skull-stripped, segmented into three kinds of brain tissues (i.e., white matter, gray matter, and cerebrospinal fluid), nonlinearly deformed into standard MNI brain space. After discarding the first five volume, the resting state fMRI datasets were preprocessed using the following steps: slice-timing correction, motion correction, skull stripping, regressing out nuisance signals (i.e., white matter, cerebrospinal fluid, and Friston-24 motion parameters) as well as linear and quantic trends, nonlinearly wrapped into standard brain space, bandpass filtering (0.01–0.1 Hz), spatially smoothing (FWHM = 6 mm). Of note, after motion correction, the mean frame-wise displacement (FD) was computed as the motion covariate for each subject. Five participants with mean FD larger than 0.5 mm were discarded, resulting in 240 subjects for diagnostic model. According to statistics, 37 subjects (37%) were combined type of ADHD. 1 subject (1%) was hyperactive/impulsive type of ADHD. 62 subjects (62%) were inattentive type of ADHD.

### Time-courses of ICNs

In this study, ten well-established ICNs were taken as spatial templates. The template ICNs were obtained using meta-analysis of the BrainMap database. The names and abbreviations of the ten ICNs were present in Table 8. The time-course of each ICN was computed using the spatial regression step of dual-regression. First, the 3D image of the corresponding ICN was extracted from the ten template ICNs. Second, the 3D image of a functional volume was extracted from the 4D resting state fMRI sequence. Third, the 3D images of ICN and functional volume were reshaped into two one-dimensional vectors. Fourth, the beta value was obtained by spatial regression between the two vectors. Finally, the time-courses of each ICN were obtained by combining the beta values along the timeline of the functional sequence. The detailed steps of spatial regression could be found in previous studies^22,49^.

**Table 8:** Names of template ICNs.

index	Names of ICNs	Abbreviations
ICN1	Medial visual network	MVN
ICN2	Occipital visual network	OVN
ICN3	Lateral visual network	LVN
ICN4	Default mode network	DMN
ICN5	Cerebellum network	CBN
ICN6	Sensorimotor network	SMN
ICN7	Auditory network	AN
ICN8	Executive control network	ECN
ICN9	Right frontoparietal network	RFPN
ICN10	Left frontoparietal network	LFPN

### Inter-ICN variability

The inter-ICN variability was based on Hilbert transform with the following procedures: 1) obtain pair-wise time-courses of ICNs; 2) apply Hilbert transform on the two time-courses; 3) obtain the instantaneous phases of each time-course; 4) compute the instantaneous phase differences between the two time-courses; 5) transform instantaneous phase differences into –pi to pi; 6) the compute the variance of the instantaneous phase differences. Finally, after looping through the ten ICNs, a vector with 10 × 9/2 = 45 features were obtained for each subject. In addition, the conventional functional connectivity (FC) and phase synchrony (PS) were computed for comparisons.

### Diagnostic model

The original features were consisted of inter-ICN variability, IQ scores, age, sex and head motion. The diagnostic model could be denoted as the following formula:

$$label=\sum _{i=1}^{45}{w}_{1i}VA{R}_{i}+\sum _{i=1}^{3}{w}_{2i}I{Q}_{i}+{w}_{3}sex+{w}_{4}age+{w}_{5}mFD+b$$

In the diagnostic model, the label denotes the diagnostic information of each subject (i.e., 1 for ADHD, 0 for normal controls). The *VAR*_*i*_ denotes the ith inter-ICN variability, and *w*_1*i*_ denotes the weight of the ith inter-ICN variability. The *IQ*_*i*_ denotes verbal IQ, performance IQ or full IQ, and *w*_2*i*_ denotes the weight the ith *IQ*_*i*_. The sex denotes the gender of subject, and *w*_3_ denotes the weight of sex. The age denoted the biological age of subject, and *w*_4_ denotes the weight of age. The mFD denotes mean frame-wise displacement, and *w*_5_ denotes the weight of head motion.

The diagnostic model was analyzed using support vector machines (SVMs), which was proposed by Cortes and Vapnik in 1995^50^. SVM was designed for classification of two classes. The advantage of SVM was solving small sample problem, nonlinear problem and high dimensional pattern recognition. The basic idea of SVM was searching an optimized hyperplane which can classify different kinds of samples. In this paper, sequential minimal optimization (SMO) was applied on training and testing datasets to search the optimized hyperplane. SMO was an iterative algorithm with fast speed to effectively solve the optimizing problems of SVMs^51^. The trick of SMO was analytically solving a set of smallest possible sub-problems instead of the original SVMs. SMO significantly improve the performance and computation times of SVMs. Moreover, SMO exhibits good performance for linear SVMs. Here, SMO with linear kernels was applied in the diagnostic model. The implementation of SMO procedure was based on WEKA, which is a popular machine learning software (www.cs.waikato.ac.nz/ml/weka). In addition, the weights of features were considered as their contributions to the diagnostic model^52^. The positive weight means increased inter-ICN variability in ADHD, while the negative weight means decreased inter-ICN variability in ADHD.

### Performance of diagnostic model

To evaluate the performance of diagnostic model, cross-validation (CV) was applied in this study. In an n-fold CV, the original samples were first divided into n-folds. Then, one-fold of samples were selected as testing samples, leaving the rest instances as training samples. Third, a diagnostic model was built on the training samples. Fourth, the trained diagnostic model was tested using testing samples. Finally, n diagnostic models were trained and tested based on n-fold CV. In this paper, leave-one-out cross-validation (LOOCV) and 10-folds CV were applied to evaluate the performance of diagnostic model. Specially, 1000 simulations of 10-fold CV were performed to validate the effects of random seeds in partitions of folds.

The performance of classification was evaluated by total accuracy (Acc.), sensitivity (Sen.) and specificity (Spec.). Here, let TP denotes the number of children with ADHD correctly classified as patients. FP denotes the number of healthy controls incorrectly classified as ADHD. TN denotes the number of correctly identified healthy subjects. FN denotes the number of incorrectly identified patients. Total accuracy is defined as the proportion of correctly predicted instances (i.e., accuracy = (TN + TP)/(TN + FN + TP + FP)). Sensitivity is the proportion of correctly classified positive instances (i.e., sensitivity = TP/(TP + FN)). Specificity is the proportion of correctly classified negative instances (i.e., specificity = TN/(TN + FP)).

### Data availability

The MRI datasets could be obtained from a public database (http://fcon_1000.projects.nitrc.org/indi/adhd200/).
